# Internationalisation of information services for publishers' open access policies: the DINI multilingual integration layer

**DOI:** 10.1186/1747-5341-3-19

**Published:** 2008-07-28

**Authors:** Frank Scholze

**Affiliations:** 1 Stuttgart University Library, 70174, Stuttgart, Germany; 2 Ministry of Science, Research and the Arts Baden-Wuerttemberg, 70173, Stuttgart, Germany

## Abstract

It is essential for the strategy of open access self-archiving that scientific authors are given comprehensive information on publisher copyright policies. DINI, the German Initiative for Networked Information, has developed a German (and potentially multilingual) interface to the English SHERPA/RoMEO service to provide additional information on German publishers' open access policies. As a next step, this interface was enhanced to an integration layer combining different sources on publisher copyright policies. This integration layer can be used in many different contexts. Together with the SHERPA/RoMEO team, DINI aims to build an international support structure for open access information.

## Background

"How long is a service giving information about Copyright Transfer Agreements (CTA) necessary?" asked Fred Friend^a ^– and gave a winking answer: "As long as there is not 100% open access or as long as not all scientific journals are owned by one of the big international publishers."

There are two primary vehicles for delivering open access (OA) for research articles, OA journals and author self-archiving in OA repositories. Authors need no permission for preprint archiving. When they have finished writing the preprint, they still hold copyright. If a journal refuses to consider articles that have circulated as preprints, that is an optional journal submission policy, not a requirement of copyright law. If authors transfer copyright in the postprint to a journal, then they need the copyright holder's permission to deposit it in an OA archive [1].

It is essential for the strategy of self-archiving to give scientific authors comprehensive information on publisher copyright policies. Therefore the RoMEO Project (Rights MEtadata for Open archiving) [2] was funded by the UK Joint Information Systems Committee (JISC) for one year (August 2002 – July 2003) to investigate the rights issues surrounding the 'self-archiving' of research in the UK academic community. The resulting service is now maintained by SHERPA, with support from JISC and the Wellcome Trust. Journal information is provided by the British Library's Zetoc service hosted by MIMAS. It mainly contains information on publishers' policies from the Science, Technology and Medicine (STM) communities in the Anglophone world. Information is given in English only. Policy information is aligned with journal titles at the publisher level, which means that it is not possible to display varied policies at the Journal level.

Therefore DINI, the German Initiative for Networked Information, conducted a short project (OA policies phase I: July 2006 – January 2007) funded by the DFG (Deutsche Forschungsgemeinschaft) to develop a German interface to the SHERPA/RoMEO service and to provide additional information on German publishers' open access policies [3]. It examined the stances of publishers in Germany as expressed in the Copyright Transfer Agreements (CTAs) of the journals towards self-archiving, and the practice of depositing digital copies of authors' works in an OAI-compliant open access repository. National language publishers in Germany are mainly active in the arts, humanities and social sciences. The interface developed in phase I of the project draws its data exclusively from the SHERPA/RoMEO service as a central and international source of journal policy information. The underlying assumption is that in parallel to the development of a translation interface, the ROMEO service can be enhanced to support online distributed inclusion of local or national journal policy data from "editorial teams" like DINI in Germany.

In the course of phase I it became obvious that, due to a lack of resources at that time, SHERPA/RoMEO could not extend the functionality of its service in order to support multiple languages or to provide online access to insert or edit national journal information. Only two options seemed possible to enhance the SHERPA/RoMEO service with additional data. One was to build an enhanced database harvesting from RoMEO (an approach followed by the Australian OAK law project discussed as related work in the following section) and the other was to build an integration layer akin to a meta search engine that could collect and process data from different sources in order to present the information in a uniform way. This approach was taken up in phase II of the DINI-DFG OA policies project (April 2007 – October 2007). However DINI strongly supports the further development of the SHERPA/RoMEO service in order to ensure that it continues to be the most prominent and encompassing source of information in the field.

### OAK List

The OAK List [4] was formally launched on February 8th 2008. It was developed and is maintained by the Law Faculty at Queensland University of Technology Brisbaine (QUT). Key features of the OAK List are:

- Information about Australian publishers and journals that are not listed in the SHERPA/RoMEO service

- Information dynamically imported from the SHERPA/RoMEO service (the SHERPA data is refreshed each night to ensure that it is always up to date)

- Inclusion of all journals listed on DOAJ (Directory of Open Access Journals)

- Provision of an API with XML output similar to the SHERPA/RoMEO service

The OAK List is a stand-alone enhanced database service which contains all relevant information on publishers' open access policies for Australia. It does however not provide any multilingual features. It also does not provide any mechanism to integrate local information into the SHERPA/RoMEO service. It can be a valuable source of data seen from the DINI perspective as long as the additional information has not been integrated into SHERPA/RoMEO.

## Results

### OA policies Phase I: German interface for the SHERPA/RoMEO service

The SHERPA/RoMEO service provides an API [5] through which all its browsing and querying functionality can be accessed. This API provides an XML output that can easily be processed by other services. The German (or potentially multilingual) interface consists of three functional entities

- German query interface which transforms and sends queries to the SHERPA/RoMEO API

- Translation of the result sets based on fixed descriptive terms in the SHERPA/RoMEO service

- Transformation of the translated results into HTML and result delivery

The resulting technical development has been in production on the DINI website since January 2007 and is accessible from . The interface is written completely in PHP. The high standardization of descriptions (though not yet fully completed) in the SHERPA/RoMEO service makes it possible to translate about 80% of its content without using any natural language processing tools. Non-standardized descriptions of publishers' conditions are simply omitted from the translation process and displayed in their original language. The code has also been given to the Open University of Spain (UNED), which is building a Spanish interface to the SHERPA/RoMEO service [6].

### OA Policies Phase II: Integration layer oaPAPI

Although the code for the German interface is being re-used by the Spanish, it is still a one-to-one language translation application which does not provide real multilingualism in terms of translating a number of different sources into one target language. Additionally, the need to provide policy information for individual journal titles (not just publishers) and the need to give information about the origin and reliability of the data itself led to the development of an extensible oaPAPI (open access Policies Application Programming Interface) which could combine the SHERPA/RoMEO database with other data sources. To achieve this, the interface uses a highly-flexible temporary internal database. Due to a nearly-unlimited possibility in the number of languages, there is an impractical number of possible fields in the database. A fixed database schema cannot be used. The database should also be able to mirror any XML-structure. Therefore the database uses only two tables: entities and nodes. An entity stores the data of one single tag, the node describes the dependencies in a 1:n-relationship. The entities table consists of the following fields:

- id

- tag

- attributes

- language

- content

- child.

**Figure 1:**
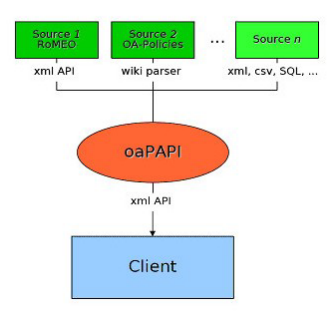


The child information improves the performance to trace back a database hierarchy. It is also an expedient way to store language information in a tag of its own instead of "attributes" to improve the search performance. The advantage for internationalization is that a real many-to-one relationship of languages is possible with this approach. In contrast to the interface developed in phase I, different translation modules can be added (e.g. English to German – which exists – plus Italian to German plus French to German) depending on the sources that are to be integrated. Efforts for writing such translation modules depend mainly on the standardization of information in the source database and on the complexity of the languages themselves. So far there is no cost evaluation for inclusion of non-Roman alphabet languages like Hebrew, Arabic, Chinese etc. The basic functional model is shown in Figure. 1.

The connector to the SHERPA/RoMEO API, which already exists, has been integrated. In addition, a WIKI parser has been added. This allows the storing of additional (e.g. German) publisher or journal title copyright information in a simple XML form which can be processed and integrated with the information from the SHERPA/RoMEO service. Currently this local information is administered in English in order facilitate a later inclusion in the Sherpa/RoMEO service. Like the RoMEO data it is translated into German on the fly using the translation module developed in phase I. Input from the Australian OAK List can be integrated accordingly through its XML interface, for example. This flexibility can be regarded as "avant-garde" for the development of the SHERPA/RoMEO service. It is DINI's objective to sustain the SHERPA/RoMEO service as the most comprehensive and global source of Publishers' Open Access policies, adding more functionality and a more complex data model in the course of time.

The oaPAPI code developed so far is available from . Due to the short duration of the second phase of the project, no user interface has yet been added. With the integration of a larger number of sources, a more sophisticated set of rules has to be implemented if there is contradictory information in a publisher or a journal open access policy. This has been left to a third phase of the project, which has just been granted funding and will start in September 2008.

## Conclusion

As open access is an international phenomenon, multilingual support for scientific authors is necessary in order for them to decide about self-archiving their scientific publications [7]. Currently support is centered on big STM publishers and available in English only. DINI provides a multilingual integration layer for different information sources which can be used in different contexts and settings. Together with the SHERPA/RoMEO team, DINI aims to build an international support structure for open access information. oaPAPI can become a tool to support this.

## Endnote

^a ^Fred Friend is JISC Consultant and Honorary Director, Scholarly Communication, University College London.

## About the Author

Frank Scholze is head of the public services department at Stuttgart University Library. Currently he is programme manager at the Ministry of Science, Research and the Arts Baden-Wuerttemberg. He chairs the DINI (German Initiative for Networked Information) working group on electronic publishing. He has published on open access and institutional repositories and he is interested in knowledge and information management in higher education.

## Competing interests

The author declares that they have no competing interests.

## Authors' contributions

FS wrote the manuscript in its entirety.
